# ﻿*Cardaminemangshanensis*, a new species of Brassicaceae from limestone landform in Southern Hunan, China

**DOI:** 10.3897/phytokeys.256.153110

**Published:** 2025-05-21

**Authors:** Ang Liu, Xiong Li, Guo-xing Deng, Jun Chen, Zan Liu, Xun-Lin Yu, Lei Wu

**Affiliations:** 1 Central South University of Forestry & Technology, Changsha 410004, Hunan, China Central South University of Forestry & Technology Changsha China; 2 Guangxi Forestry Inventory and Planning Institute, Nanning 530011, Guangxi, China Guangxi Forestry Inventory and Planning Institute Nanning China; 3 Hunan Mangshan National Nature Reserve Management Bureau, Yizhang 424221, Hunan, China Hunan Mangshan National Nature Reserve Management Bureau Yizhang China

**Keywords:** *Cardamine*, limestone landform, new species, taxonomy

## Abstract

*Cardaminemangshanensis*, a new species, is described and illustrated from the limestone landform in southern Hunan, China. The new species is similar to *C.macrophylla*, but differs from the latter in having more prominent tubers, shorter plant height (10–25 cm vs. 30–95 cm), fewer cauline leaves (3–4 vs. 3–18), crenate leaf margin (vs. serrate), fewer flowers (4–10 vs. 10–30), an earlier flowering period (late February to mid-March vs. April to June) and a shorter growth cycle (ca. 4 months vs. ca. 8 months). Following the IUCN Red List Criteria, *C.mangshanensis* is assessed as ‘Vulnerable, VU B2ab(ii)’.

## ﻿Introduction

The genus *Cardamine* L. comprises approximately 300 species (https://cardamine.sav.sk), distributed on all continents except Antarctica. According to the Flora of China (Zhou et al. 2001), 48 species have been recorded in China, of which 24 are endemic. Since then, several new species of *Cardamine* from China have been described, including *C.hunanensis* (Wu et al. 2021), *C.libagouensis* (Diao et al. 2023), *C.sichuanensis* (Guo et al. 2024), *C.zhangjiajieensis* (Li et al. 2024), and others. Notably, some species within this genus are valued for their edible qualities. For example, in certain regions of southern China, the tender leaves of *C.macrophylla* Willd. are highly prized by locals for their delicate flavor.

On 25 March 2020, during an investigation in Mangshan National Nature Reserve, we discovered a unique species of the genus *Cardamine*. This plant had already passed its peak flowering period, with ovaries developing into nascent fruits, indicating that it was different from the plants of the same genus recorded in Hunan Province (Qi and Yu 2002; Wu et al. 2021; Li et al. 2024). To further study this unique discovery, we returned to the same site for an investigation the following year, one month in advance. On 27 February 2021, we successfully collected specimens in flowering season as planned. However, when we returned in mid-April, despite arriving half a month earlier than the previous year, we encountered the same situation: the plants had already entered a state of decay. This prevented us from observing mature fruits and seeds. Nevertheless, based on our comprehensive understanding of its morphological characteristics and phenological patterns, we confidently conclude that this represents a new species.

## ﻿Material and methods

The specimens are primarily deposited in the Herbarium of Forest Plants at Central South University of Forestry and Technology (CSFI). Morphological observations of the new species were derived from field observations and examination of herbarium specimens. Comparative analyses of related species were examined using online images from JSTOR Global Plants (https://plants.jstor.org/). The conservation status of the new species is based on field observations in accordance with IUCN Red List guidelines (IUCN 2024).

## ﻿Taxonomic treatment

### ﻿ Cardamine mangshanensis

Taxon classificationPlantaeBrassicalesBrassicaceae

X.L.Yu, A.Liu & X.Li sp. nov.

DED00102-7E8A-51AB-88F7-45C6D83341AB

urn:lsid:ipni.org:names:77362019-1

Figs 1 , 2

#### Diagnosis.

This new species is similar to *C.macrophylla* Willd., but it differs in the following characteristics: more prominent tubers, shorter plant height (10–25 cm vs. 20–115 cm), fewer cauline leaves (3–4 vs. 3–18), distinctly petiolulate leaflets (vs. sessile or occasionally with a short petiole), crenate leaf margin (vs. serrate), fewer flowers (4–10 vs. 10–30), white flowers (vs. purple or lilac, rarely white), an earlier flowering period (late February to mid-March vs. April to June) and shorter growth cycle in one year (ca. 3 months vs. ca. 8 months) (See Fig. 4, Table 1).

**Table 1.** Comparison of morphological characters between *Cardaminemangshanensis* sp. nov. and *C.macrophylla*.

Characters	*Cardaminemangshanensis* sp. nov.	*C.macrophylla*
Plant	10–25 cm high	(20-) 30–95 (-115) cm high
Rhizome	with obvious tubers	with tuberous knots
Cauline leaves	3–4	3–18
terminal leaflet	petiole 0.5–1.5 cm long	sessile
lateral leaflets	2–4 pairs	2–7(-11) pairs
	petiole 0.5–2.5 cm long	sessile or occasionally with short petiole (no more than 1 cm)
	margin crenate	margin serrate
	base obliquely cordate	base cuneate or obliquely decurrent
Racemes	4–10 flowers	10–40 flowers
Flower color	white	purple or lilac, rarely white
Flowering period	late February to mid-March	April to June
Growth cycle in one year	ca. 3 months	ca. 8 months

#### Type.

China. Hunan: Chenzhou City, Yizhang County, Mt. Mangshan, Hejiawan, in sparse forests within limestone areas, elevation ca. 650 m, 27 February 2021, *Ang Liu & You-ke Gong* HJW01 (Holotype CSFI!, isotype CSFI!, HIB!&CSH!) (See Fig. 3).

**Figure 1.**
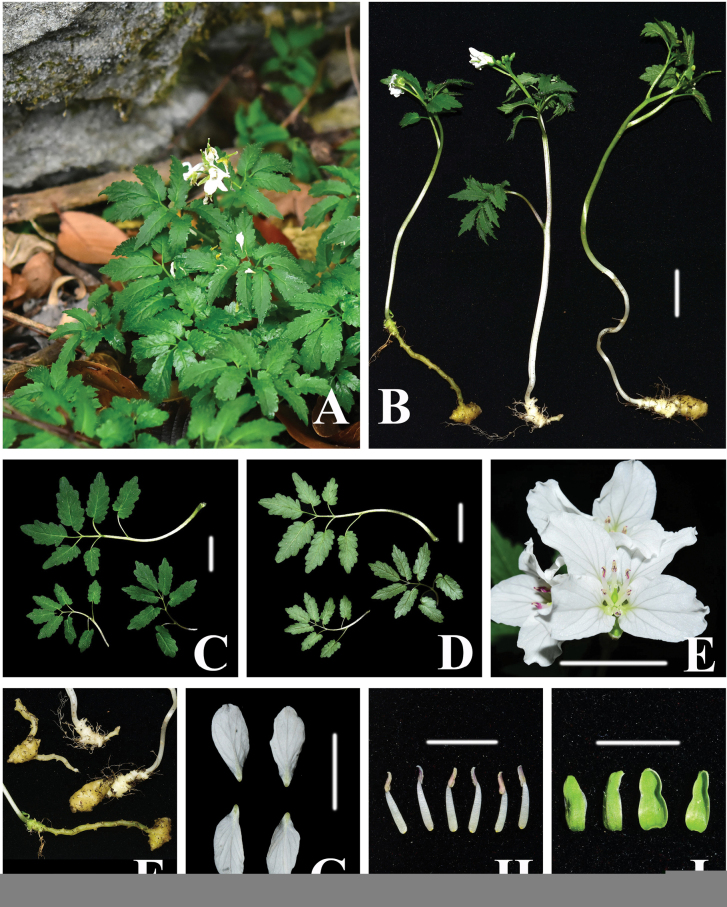
*Cardaminemangshanensis* sp. nov. **A** habit **B** plants **C** ventral view of leaves **D** dorsal view of leaves **E** front view of flowers **F** rhizomes **G** petals **H** stamens **I** sepals. Photographs by Ang Liu. Scale bars: 1 cm.

**Figure 2.**
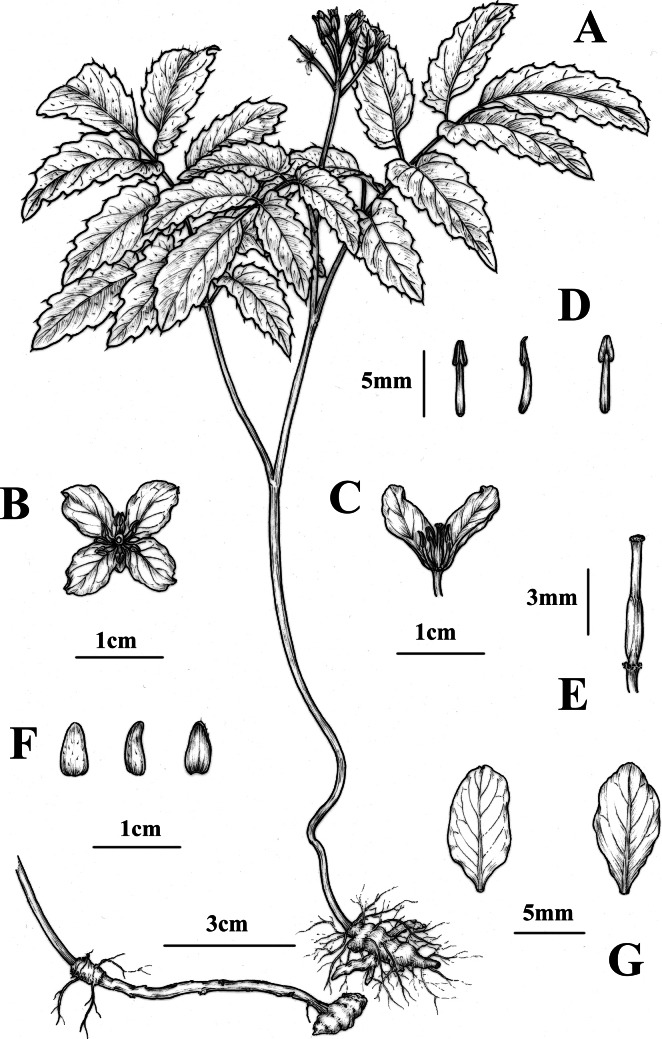
*Cardaminemangshanensis* sp. nov. **A** plant and rhizomes **B** top view of flower **C** longitudinal section of flower **D** stamens **E** pistil **F** sepals **G** petals. drawn by phd jing tian; based on the holotype: *ang liu & you-ke gong* hjw01, csfi 076289 and living plants from type locality.

**Figure 3.**
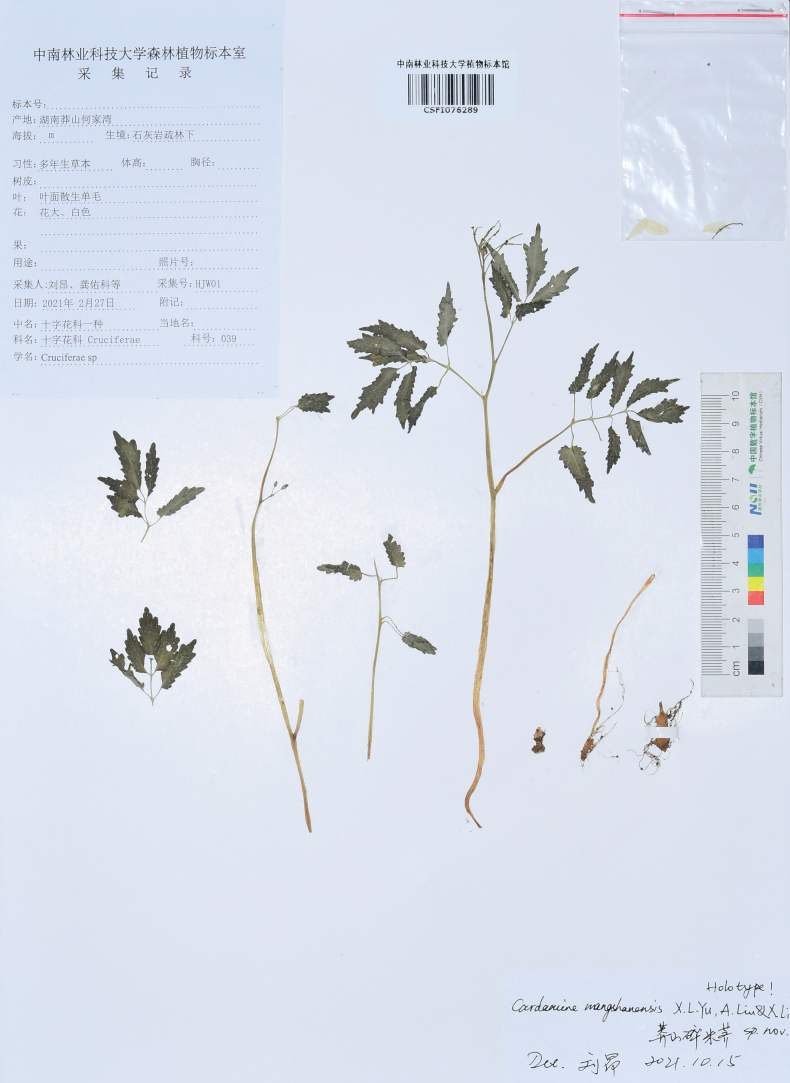
Holotype of *Cardaminemangshanensis* sp. nov. (*Ang Liu & You-ke Gong* HJW01, CSFI 076289).

**Figure 4.**
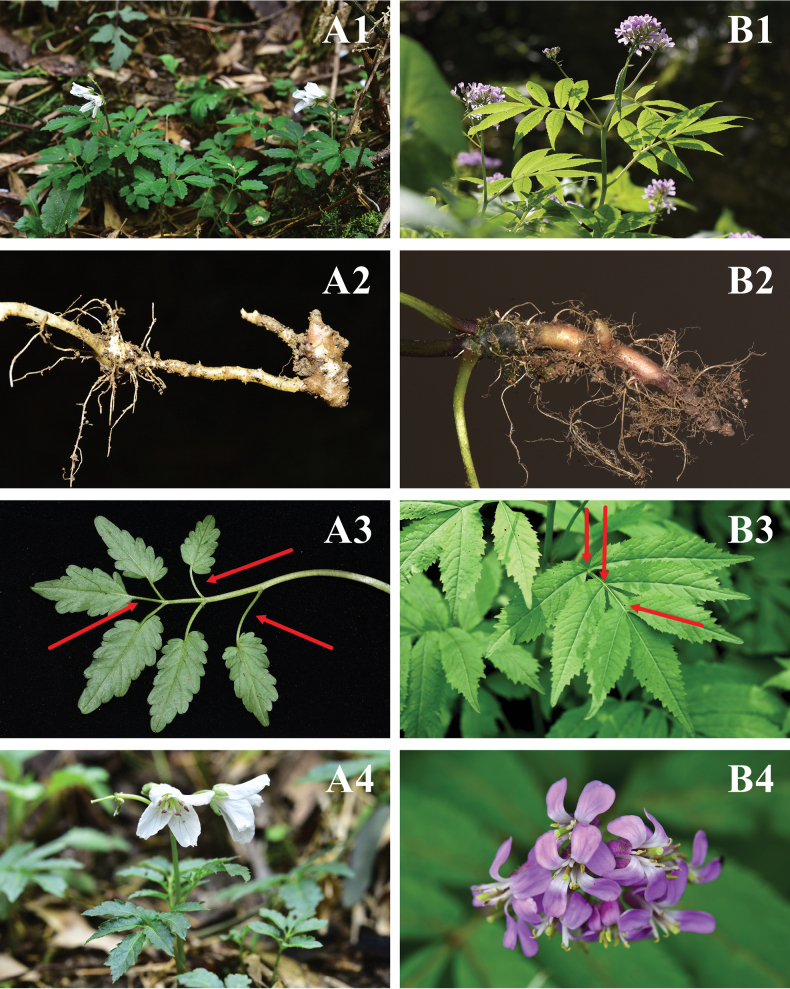
Morphological comparison of *Cardaminemangshanensis* sp. nov. (**A1–A4**) and *C.macrophylla* (**B1–B4**) **A1, B1** plant **A2, B2** rhizome **A3, B3** Leaf **A4, B4** flowers. Photographs by Ang Liu.

#### Description.

Herbs perennial, delicate, 10–25 cm high. ***Rhizomes*** creeping, with tubers, 0.3–5 cm long. ***Stems*** erect, unbranched, surface furrowed, smooth. ***Cauline leaves*** 3–4, petiole 1.5–5 cm long, smooth, not auriculate at base, margin glandular serrate; ***terminal leaflets*** elliptic or oblong, (1.5-) 2.5–4 (-5.5) × (0.5-) 1–2.5 (-3) cm, sparsely pilose, petiole 0.5–1.5 cm long, base cuneate, margin crenate, rarely doubly serrate, apex acute, slightly caudate; ***lateral leaflets*** 2–4 pairs, sparsely pilose, petiole 0.5–2.5 cm long, base obliquely cordate or truncate, margin crenate, equal to or slightly smaller than terminal leaflets. ***Racemes*** 4–10 flowered, dense, inflorescence slightly elongated in fruit, pedicel 0.5–1.5 cm long. ***Sepals*** elliptic, ca. 0.5 cm long, green, glabrous or very sparsely pilose. ***Petals*** white, obovate, 1 cm long, blunt at the top and cuneate at the base. ***Stamens*** glabrous, ca. 6 mm long, filaments about 4 mm long, base slightly dilated, anthers purplish red. ***Pistils*** 5–7 mm long, ovary columnar, style slender, ca. 3 mm long, equal to or slightly longer than the ovary. Mature fruit not observed.

#### Phenology.

Flowering occurs from late February to mid-March. Interestingly, after completing seed propagation (before mid-April), the aboveground parts of the plant wither.

#### Etymology.

The specific epithet of this new species is derived from its type locality, Mt. Mangshan, one of the most famous mountains in southern Hunan.

#### Distribution and habitat.

This new species is currently known only from the limestone landform area of Mt. Mangshan, where it typically grows beneath sparse bamboo forests.

#### Additional specimens examined

**(*Paratypes*).** China. Hunan: Chenzhou City, Yizhang County, Mt. Mangshan, Hejiawan, in sparse forests within limestone areas, elevation ca. 650 m, 25 March 2020, *Ang Liu & Xiong Li* LAHJW01 (CSFI!, HIB! & CSH!).

#### Conservation status.

Currently, only one population with a total of about 300 individuals has been found in the limestone areas of Mt. Mangshan. This population is located within the boundaries of Mangshan National Nature Reserve and is minimally affected by human activities. Based on the IUCN Red List criteria (IUCN 2024), the conservation status of the new species should be better categorized as ‘Vulnerable, VU B2ab(ii)’.

## ﻿Discussion

**Figure 5.**
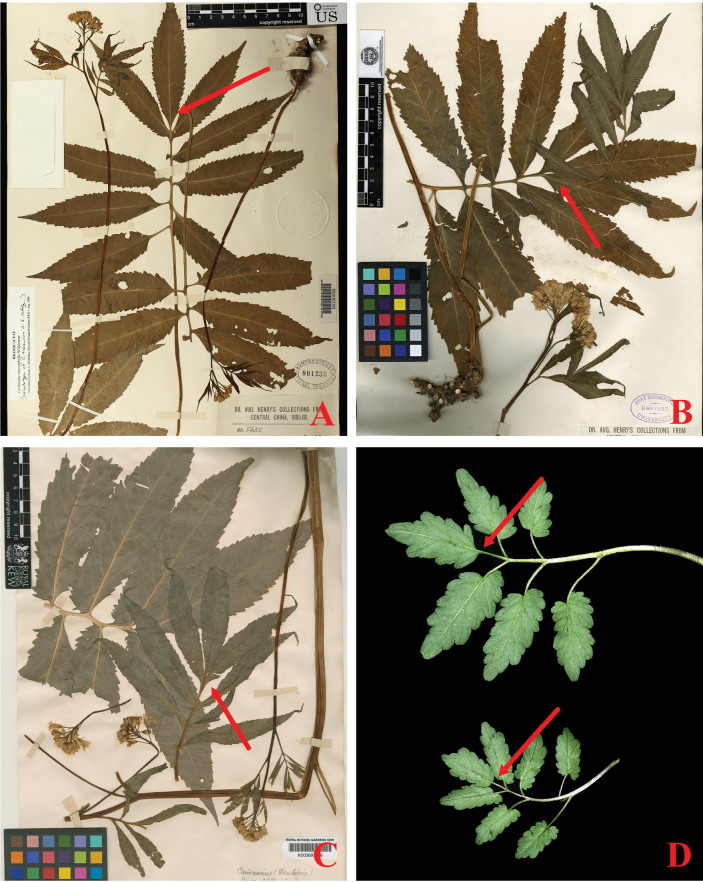
Leaf morphological variation of *Cardaminemacrophylla* (**A–C**) and *C.mangshanensis* sp. nov. (**D**) **A** Henry 5635(GH00112001) **B** Henry 5635(US00100058) **C** Henry 5635 (K000697746) **A–C** from JSTOR (https://plants.jstor.org/) **D** from type locality of *C.mangshanensis*, photograph by Ang Liu.

The Flora of China notes that *C.macrophylla* exhibits a considerable degree of variability, particularly in terms of leaflet number, shape, size, basal structure, and margin characteristics. Special attention is given to the specimen cataloged as Henry 5635, which serves as a key reference in this context. Similar phenomena have been observed in our field studies. However, regardless of the variation in the length of the petioles of the lateral leaflets, the terminal leaflet and the pair of lateral leaflets directly beneath it consistently lack petioles (See Fig. 5A–C). It is also evident that the terminal leaflet is positioned in close proximity to the pair of lateral leaflets below it. Through extensive field research and specimen analysis, we have confirmed that the presence of petioles in the lateral leaflets of *C.macrophylla* is an individual variation rather than a universal trait. Importantly, the terminal leaflet never has petioles.

However, the terminal leaflet of *C.mangshanensis* is clearly petiolulate, measuring 0.5–1.5 cm in length. Additionally, the pair of lateral leaflets on its lower side are clearly not closely attached and has petioles that are 0.5–2.5 cm long (See Figs 4A3, 5D). The differences in these traits all prove that *C.mangshanensis* does not belong to the *C.macrophylla* complex, but is a new species.

Based on our observations, we have identified a significant correlation between the leaf size of this species and annual precipitation levels. In years with abundant rainfall, such as 2020, the leaves exhibit a marked increase in size, measuring approximately 3–5.5 cm in length and 1.5–2.5 cm in width. In contrast, during years with limited rainfall, such as 2021, the leaves are significantly smaller, ranging from 1.5–2.5 cm in length and 0.5–1 cm in width. This adaptive response to environmental fluctuations highlights the species’ enhanced reproductive capacity and ecological resilience.

These findings provide deeper insights into the complex relationship between morphological traits and environmental factors in *C.mangshanensis*, and underline its remarkable ability to thrive under varying climatic conditions in limestone landform.

## Supplementary Material

XML Treatment for Cardamine mangshanensis

## ﻿Additional information

### Author ORCIDs

Ang Liu https://orcid.org/0000-0001-6281-7145

Lei Wu https://orcid.org/0000-0003-1451-7855

### Data availability

All of the data that support the findings of this study are available in the main text.
